# Cochlear implantation in vestibular schwannoma: A game changer? A narrative review

**DOI:** 10.1016/j.bjorl.2025.101563

**Published:** 2025-02-05

**Authors:** Luis Lassaletta, Miryam Calvino, Isabel Sánchez-Cuadrado, José Manuel Morales-Puebla, Javier Gavilán

**Affiliations:** a Hospital Universitario La Paz, IdiPAZ Research Institute, Department of Otorhinolaryngology, Madrid, Spain; b Biomedical Research Networking Centre on Rare Diseases (CIBERER), Institute of Health Carlos III, Madrid, Spain; c Universidad Autónoma de Madrid, Madrid, Spain

**Keywords:** Vestibular schwannoma, Hearing loss, Cochlear implantation, Neurofibromatosis type-2, Auditory Nerve Test System (ANTS)

## Abstract

**Objectives:**

In recent years, indications for Cochlear Implants (CIs) have been on the rise, with Vestibular Schwannoma (VS) patients among these emerging indications. On the other hand, there is growing evidence in favor of the 'wait and see' approach as opposed to surgery or irradiation for most small VS. The aim of this paper is to discuss the current role of CIs in patients with VS, addressing the most controversial topics such as the variability of outcomes, the emerging clinical scenarios, the lack of consistent prognostic factors, and the need for an intraoperative tool to aid decision-making in challenging cases.

**Methods:**

A Pubmed search was performed, with a selection of relevant citations, and critical appraisal of selected studies. Articles written in English on VS and cochlear implantation were eligible for inclusion. Pictures of illustrative cases from a tertiary center were included.

**Results:**

The discussed topics were the following: (1) Variability in audiological outcomes; (2) Prognostic factors including tumor size, preoperative hearing, previous irradiation, extent of resection, and Neurofibromatosis type II **(**NF2) status; (3) Postoperative imaging; (4) The role of intraoperative testing; and (5) The current indication of CIs in different clinical scenarios.

**Conclusions:**

CIs have become a useful solution for certain patients with VS. Audiological outcomes are variable, but worse than those of conventional CI candidates. Small and medium-sized tumors and those with a better preoperative hearing are more likely to benefit from a CI. The effect of irradiation is uncertain. Outcomes in NF2 patients may decrease in the long term. The auditory nerve test system is a useful intraoperative tool in most scenarios. The variability of the outcomes still precludes routine CI in VS with normal contralateral hearing. In addition to VS undergoing surgical resection and CI placement, some observed and irradiated VS cases also benefit from a CI.

**Level of evidence:**

5.

## Highlights

• Cochlear implantation is a solution for some patients with vestibular schwannoma.
• Audiological outcomes are variable, but worse than conventional CI candidates.
• Cochlear implantation can be performed in operated, observed or irradiated cases.
• The Auditory Nerve Test System is a useful intraoperative tool.
• A positive wave V elicited with the ANTS strongly predicts at least sound detection.

## Introduction

Indications for Cochlear Implants (CIs) have significantly expanded. Initially, only individuals with profound bilateral Sensorineural Hearing Loss (SNHL) of cochlear origin were considered. In recent years, the range of indications has steadily grown. Among these indications is the possibility of implanting patients with Vestibular Schwannoma (VS); that is, patients with SNHL of retrocochlear origin.^1^ Initially, this was explored as an alternative to Auditory Brainstem Implants (ABIs) in patients with Neurofibromatosis type 2 (NF2). More recently, cochlear implantation has been considered even for patients with sporadic tumors with normal contralateral hearing.^2^

On the other hand, the management of patients with VS has also evolved over the years. While some decades ago, surgical resection was often the default option for most patients, our understanding of the growth rate and behavior of these benign lesions has led to an increase in conservative management cases.^3^, ^4^

Irrespective of these considerations, most patients with VS will suffer from hearing loss. The three therapeutic options, either observation, surgery or irradiation, usually lead to hearing loss in the long term.^5^ In this new and conservative paradigm regarding the approach to patients with VS, CIs have emerged strongly as a possibility for compensating hearing loss in certain cases.

However, there are still several controversial topics surrounding cochlear implantation in patients with VS. Some of these include:

1) Variability in audiological outcomes: What can we expect from placing a CI in a VS patient?
2) Are tumor size, preoperative hearing, previous irradiation, extent of resection, and NF2 status consistent prognostic factors?
3) Can Magnetic Resonance Imaging (MRI) safely be performed in patients with CI? Can we control the ipsilateral Internal Auditory Canal (IAC) if a CI is placed?
4) Is there a need for intraoperative testing?
5) What are the current VS clinical scenarios in which a CI can be considered? Having CI as a potential treatment for hearing loss, change the overall approach to patients with VS?

The focus of this revision is on the insights gained from the extensive experience of a tertiary center in treating patients with VS considering CI in different scenarios. Representative cases from our institution are included to illustrate these controversial topics.

## Headings

### Global outcomes. Variability

As the functionality of the Cochlear Nerve (CN) may be compromised in various ways, the outcome of cochlear implantation in VS patients is variable. Different studies have shown speech intelligibility scores ranging from 37% to 72% one year post-implantation.^1^, ^6^, ^7^ Dornhoffer et al.^2^ reviewed 49 VS patients undergoing cochlear implantation both sporadic (n = 21) or NF2 (n = 28). About half the patients who underwent microsurgery achieved open-set speech recognition. Butler et al.^8^ found that 50% of patients who underwent simultaneous tumor resection with cochlear implantation (n = 46) demonstrated open-set speech abilities, while 80.4% reported daily CI use. While these may be encouraging outcomes, especially for NF2 patients, they are still far from those obtained in conventional CI candidates.^9^ Therefore, it is imperative to adjust the patient’s expectations. This variability in the outcomes may be explained by several variables as tumor size, preoperative hearing, irradiation, NF2 status or the degree of tumor resection in surgical cases.

### Prognostic factors

#### Size

As a general rule, only patients with small or medium size tumors are good candidates to preserve a functional CN and therefore undergo cochlear implantation following tumor resection. In a systematic review, Wick et al.^10^ reported a mean tumor size of 1.2 cm in 46 patients from 20 studies.

However, a size limit has not been established to resect a VS sparing the functionality of the CN. Not only the size but also the adherence of the tumor to the CN has an impact on the nerve preservation probability, and this feature is still unpredictable.

Interestingly, resection of some large tumors with preservation of the CN and a successful CI have been reported, sometimes the measurement including an associated cystic component.^11^ As an example, Aristegui et al.^12^ reported a successful CI in a Translabyrinthine (TL) approach for a 4 cm multicystic VS which is still working nowadays (unpublished data). The solid component of the tumor was much smaller than the total size.

#### Preoperative hearing

CIs have traditionally been indicated for patients with severe-to-profound SNHL of cochlear origin, with the specific audiological criteria for indication becoming less stringent in recent years. As an emergent CI indication there are less regulations and the literature shows a large variation of hearing indications for patients with VS, some studies including patients with ipsilateral serviceable hearing.^13^, ^14^

The exact mechanisms behind hearing loss caused by VS remain unclear. Potential factors include compression of the CN, vascular compromise of the internal auditory artery or the vasa nervorum, and biochemical changes in the inner ear fluids.^15^ Consequently, the specific contributions of cochlear and retrocochlear elements of hearing loss, whether due to the tumor itself or its treatment (observation, radiation therapy, or surgery), on the CN's ability to transmit electrical signals are still not fully understood.

Not surprisingly, a better preoperative hearing may be related to a better outcome postimplantion as it may lead to a better functioning CN.^16^ In a systematic review conducted by West et al.^7^ involving 86 VS patients, preoperative hearing was identified as a significant prognostic factor. Among those with preoperative hearing classified as class A or B, 78% demonstrated high postoperative performance. This contrasts with only 51% of patients whose preoperative hearing was classified as class C or D. Similarly, Sanna et al.^17^ also reported that better preoperative hearing was associated with more favorable outcomes in resected VS cases followed by simultaneous CI.

Irrespective of the therapeutic option combined with a CI, a better preoperative hearing suggests a better functioning CN. This finding supports a proactive attitude towards restoring hearing with a CI before hearing is completely lost. Again, there is no defined cutoff hearing level from which a CI would be indicated. Also, the impact of other factors as the duration of profound hearing loss or the impact of contralateral hearing is uncertain.

#### Irradiation

The impact of irradiation on CI outcomes, whether used as the primary treatment for VS or administered prior to surgery, remains a subject of debate.

**Fig. 1:**
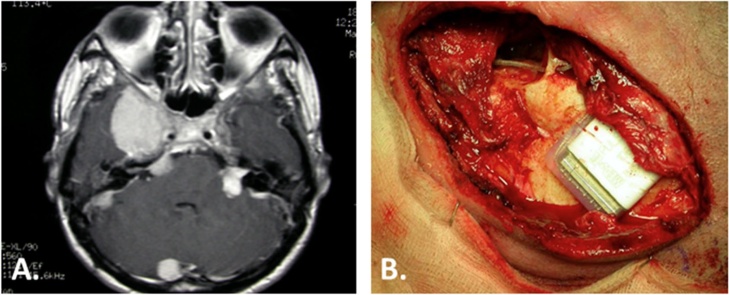
Progressive deterioration of hearing with the CI following irradiation. A NF2 27-year-old woman with multiple tumors (A) and bilateral hearing loss underwent irradiation to the left VS in 1999 and suffered a sudden hearing loss in 2004 with no recovery. A left CI (B) was placed 2 years later. Initially she achieved sound detection which aided lip reading with even some closed-set discrimination. She used the implant daily for two years. Then, the charge levels increased gradually until she had no benefit from the CI and was explanted in 2009. As no tumor growth was seen during this time, the performance decrease was attributed to the late effect of irradiation.

On one hand, some studies suggest that results of CIs are similar in both irradiated and non-irradiated patients.^18^, ^19^ On the other hand, other studies have reported irradiation as a negative prognostic factor.^1^, ^20^ Fig. 1 shows a case with a decrease in the CI performance due to irradiation.

There are different irradiation strategies and schemes. The precise mechanism why hearing is impaired following irradiation is unknown and may by multifactorial. To explain the lack of worse outcomes following irradiation in some cases it has been postulated that radiation-induced injury takes place at the level of the stria vascularis, and a CI can bypass this condition.^21^ Although one of the most crucial aspects in terms of hearing preservation is the amount of radiation delivered to the cochlea, the effect of irradiation on the CN may be unpredictable. Therefore, it may be too simplistic to just consider previous irradiation as a dichotomic variable having or not an impact on the CI outcome. Other variables as strategies, total radiation dosage, preoperative hearing, duration of deafness, and interval from treatment to CI placement have to be considered when assessing the effect of irradiation on CI outcomes.

#### Extent of surgical resection

In order to spare both the functionality of the facial and the CN, the possibility of less-than-total resection may be considered in certain VS candidates to cochlear implantation. A near-total resection was described in a VS patient undergoing CI with a simultaneous resection using a TL approach.^22^ As mentioned earlier, CI may be even considered in observed tumors in which further growth cannot be completely ruled out. While a subtotal resection may increase the chances of preserving the CN function, it may also lead to recurrence or growth of the residual tumor which may impair the CI outcome in the long term, especially in NF2 cases which are more prone to recurrence and tumor growth. Therefore, a balanced and individualized decision should be taken depending on tumor size and growth rate, age, hearing status, and patients’ preferences.

#### NF2 status

**Fig. 2:**
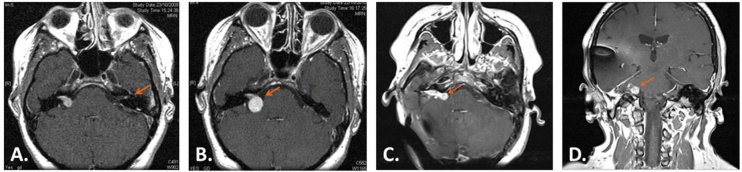
NF2 patient who underwent right CI placement due to a growing tumor. A patient was sent to our institution with a left 1 cm VS in 2010. A careful revision of the imaging showed a contralateral 2 mm tumor that had been missed in the initial evaluation (A, orange arrow shows a 2 mm left VS). After a one-year of follow-up the right left tumor had grown 5 mm and hearing had deteriorated significantly (B, orange arrow shows tumor growth of the right VS). So in 2011, she underwent a right TL approach with gross total tumor resection and simultaneous placement of a CI. She was a happy user of her CI during 6 years until hearing decreased and a recurrence was seen despite the CI artifact (C and D show the tumor recurrence which can be easily identified despite the CI artifact). The CI was removed in 2017, and the tumor recurrence was totally resected, but the CN could not be preserved.

Whether NF2 patients have worse CI outcomes than sporadic cases remain unconclusive in the literature. Doyle et al.^16^ reported similar audiological results comparing sporadic and NF2 cases. In the systematic review performed by West et al.,^23^ both groups of patients had comparable results despite differences in tumor size, location and surgical approach. In a large retrospective study including 64 NF2 patients undergoing CI and different treatment modalities, Smith et al.^24^ reported a decline in the last follow-up (median of six years) of all audiometric scores. In a recent review by Grenier et al.^25^ about CI in patients with NF2 following different treatments, at the last follow‐up auditory performance had decreased in half of the initial CI users. The authors found no clear reasons to explain this decrease in the outcome, tumor size and irradiation having no significant impact on the final outcome. Fig. 2 shows an NF2 patient showing tumor recurrence following total resection and CI placement. The present case as well as the literature suggest that having NF2 may be a negative prognostic factor in the long term.

### Imaging

**Fig. 3:**
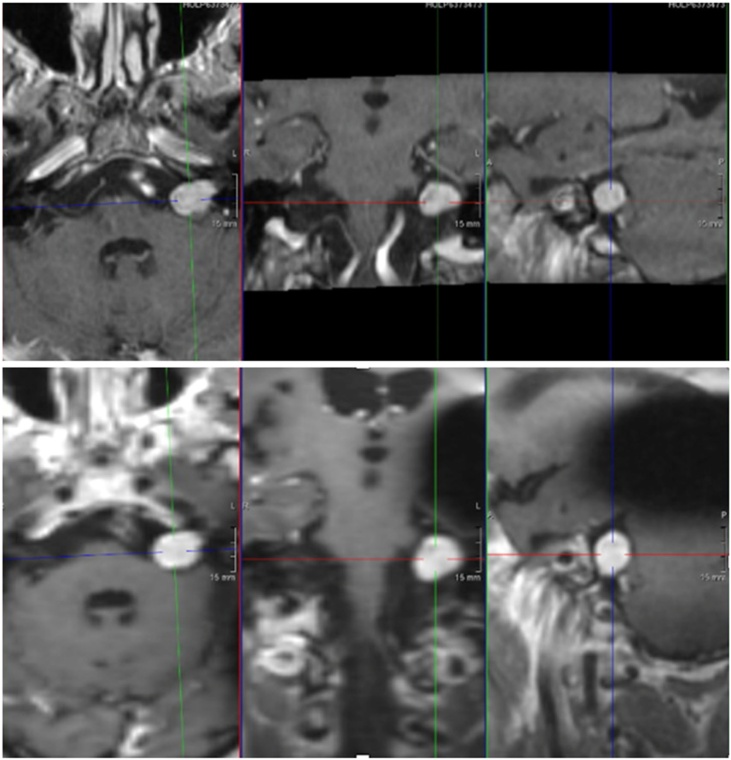
Cochlear implantation in a patient with bilateral hearing loss and a stable left VS. A 69-year-old received a left CI with no tumor removal. Above, MRI performed before cochlear implantation shows a 14 mm VS. Below, MRI performed following cochlear implantation. Note there is a black artifact which does not interfere with the correct vision of the tumor in the 3 planes (axial, coronal and sagittal).

One of the usual concerns when considering CI in patients with VS is the need for serial MRI controls which can be affected by the CI artifact. Especially, if the ipsilateral IAC can be visualized, as most VS patients will need many years of MRI follow-up. Dornhoffer et al.^2^ have suggested that “patients undergoing observation may wish to confirm a stable tumor early after diagnosis before implantation, as distortion may make surveillance challenging”. While in the early days magnet removal was advocated in order to perform brain MRI in certain patients, modern implants are MRI-compatible and using specific artifact reduction sequences is usually possible to control the ipsilateral IAC and to rule out tumor recurrence or tumor growth.^26^, ^27^ Fig. 3 shows preop and postop MRI of a patient undergoing cochlear implantation. The CI artifact does not affect visualization of the tumor.

### Variability: need of intraoperative testing

The variability in outcomes and absence of strong predictive factors emphasize the need for an intraoperative test in order to decide if placing or not a CI in a particular VS patient. There are different tools to evaluate the functionality of the CN including promontory stimulation testing, Auditory Brainstem Response (ABR), Cochlear Nerve Action Potential (CNAP), evoked compound action potentials (eCAP), or electric Dorsal cochlear Nucleus Action Potential (DNAP). However, the most reliable tool has been proved to be eABR using an intraoperative test electrode. The so-called ANTS, Auditory Nerve Test System, was developed by Med-El (Innsbruck, Austria)^28^ and initially tested by Lassaletta et al.^29^ in a subset of conventional CI candidates proving the viability of this disposable device. Since then, this intraoperative tool has been used in multiple scenarios, mostly patients with unilateral or bilateral VS.^11^

**Fig. 4:**
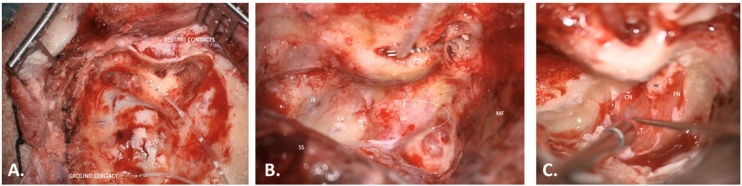
Left translabyrinthine approach for simultaneous tumor resection and cochlear implantation. (A) The test electrode is shown with its three active electrodes, and one ground electrode. (B) The test electrode is inserted in the cochlea through a posterior tympanotomy. To maintain the test electrode in place during the approach a titanium clip is used. (C) Both the Facial Nerve (FN) and the Cochlear Nerve (CN) are identified and preserved. MF, Middle Fossa; JB, Jugular Bulb; SS, Sigmoid Sinus; IAC, Internal Auditory Canal; CA, Cochlear Aqueduct; T, Tumor; HC, Horizontal Crest.

The test electrode has three active contacts which are placed inside the cochlea and one ground electrode which is placed under the temporal muscle (Fig. 4). A positive wave V elicited with the ANTS intraoperatively indicates a functional CN and supports the placement of a CI.

Lassaletta et al.^30^ have recently reported on seven VS patients. The ANTS was positive in four cases, negative in two cases, and uncertain in one case. In the five patients undergoing CI, sound detection was present. The authors include a revision of the reported studies using the ANTS for CI candidacy yielding 35 patients with complete information about eABR response. There was one false negative and one false positive case; that is, the 28 implanted cases with a present wave V following tumor resection had some degree of auditory perception in all but one case.

The main advantage of the ANTS is being an intraoperative disposable tool. The surgeon will decide if placing or not the real CI depending on the ANTS electrical response among other factors. This avoids the use of the real CI for the testing and leaving a useless device if no electrical response is elicited.

However, it still has some drawbacks. Contrary to the simplicity of other tests as eCAP which is routinely performed in every CI case, there is a learning curve for the ANTS, which includes interpreting the eABR waves and artifacts. It is not available worldwide and requires specialized trained personal. In experienced centers the reported number or false positives and negatives is very low, aiding the final decision to implant or not a particular case.

The use of the Med-El titanium clip to maintain the test electrode in place facilitates the measuring process (see Fig. 4). The main disadvantage of this system is the lack of correlation between the eABR features and the long-term hearing outcomes. While there is a strong association between obtaining a robust wave V and achieving sound detection with the implant, long-term hearing outcomes are extremely variable.

### What are the current VS clinical scenarios in which a CI can be considered? Having CI as a potential treatment for hearing loss changes the overall approach to patients with VS?

CI were initially considered for NF2 patients with bilateral profound hearing loss as an alternative to ABI.^12^, ^31^ The rationale of the procedure is to preserve a CN that will not provide natural hearing but is able to transmit the electrical pulses of the implant to the auditory pathway. Due to the usually poor outcomes of ABI in NF2 patients, it was usually stated that “the worst outcome with a CI is usually better than the best outcome with an ABI”. While this may not be accurate in every case, it reflects the worse tonotopicity and stability of ABI when compared to CI.^32^ Therefore, whenever the CN could be anatomically preserved following tumor resection in NF2 patients, a CI was considered.

Later, patients with bilateral hearing loss due to a sporadic VS in one side and a contralateral different condition leading to hearing loss were also considered as CI candidates.^1^, ^33^ In this unusual condition, there may be some controversy about which side should be implanted first, the tumor or the non-tumor side. The duration of hearing loss as well as the etiology are the key aspects in order to decide this.

In recent years, the indications for CI in VS have followed that of non-tumor CI indications, from profound bilateral hearing loss to Single-Sided Deafness (SSD).^34^, ^35^ However, there are relevant differences between tumor and non-tumor SSD cases in terms of results and satisfaction.

The main question that arises is whether having CI as a potential treatment for hearing loss changes the current approach to patients with VS. Specifically, whether smaller tumors may be over-treated with surgery or irradiation, considering that hearing can hypothetically be restored with a CI.

On one hand, a good contralateral hearing has been associated with poor audiological outcomes. Sorrentino et al.^13^ reported contralateral hearing to be a strong negative prognostic factor in a series of eight sporadic and nine NF2-associated VSs undergoing cochlear implantation. All the non-users in this series (one sporadic and three NF2 cases) had a preoperative contralateral hearing class A.

On the other hand, the clinical setting may have a strong impact in terms of patient’s satisfaction. For example, a relatively poor CI outcome (i.e., positive sound recognition with 10% open-set discrimination) may significantly improve the quality of life of a bilaterally deaf NF2 patient. However, the same audiological outcome may be useless for an implanted patient with a sporadic VS if contralateral hearing is normal.

Therefore, while CI is a useful device to improve hearing in certain VS patients it is questionable if it should be offered systematically if they have normal contralateral hearing. Moreover, in the authors’ opinion, the possibility of placing a CI following VS resection should not increase the indications for resecting small VSs if a conservative approach is acceptable.

Cochlear implantation in patients with VS can be also considered without tumor resection, both following observation and irradiation. In a study by Dornhofer et al.^2^ of a cohort of 49 patients receiving 52 CIs, patients undergoing microsurgery experienced poorer CI performance than those undergoing observation or radiosurgery, regardless of NF2 status. Lassaletta et al.^30^ have recently reported on two NF-2 and five sporadic VS, the initial scenario being simultaneous tumor resection and CI in three cases while a CI placement without tumor resection was planned in four cases. When CI is planned without tumor resection it is unclear how long the tumor should be stable, and the possibility of hearing decrease should be noted.

## Conclusions

CI is a useful solution to restore hearing in certain patients with VS, their audiological results still showing a large variability when compared to those of conventional CI candidates.

Although a size limit has not been established to resect a VS sparing the functionality of the CN, small tumors and those with a better preoperative hearing are more likely to benefit from a CI. The effect of irradiation on CI outcomes is uncertain as its effect on the CN may be unpredictable. A less-than-total resection may improve the hearing outcomes, but it may also increase the risk of tumor recurrence. Long-term CI results appear to be worse in NF2 patients. Most modern CIs are MRI-compatible and using specific artifact reduction sequences is usually possible to control the ipsilateral IAC. In addition to surgery, some observed and irradiated VS cases may also benefit from a CI. The variability in the outcomes emphasizes the need for an intraoperative tool, the ANTS having shown the best accuracy to date. While CIs have become a good alternative to ABI for patients with NF2 and patients with bilateral hearing loss, they should be indicated cautiously in those with normal contralateral hearing. Moreover, their use should not change the therapeutic approach to patients with small VS, which are better treated conservatively.

## Declaration of competing interest

The authors declare no conflicts of interest.
